# Scaffolding Strategies for Tissue Engineering and Regenerative Medicine Applications

**DOI:** 10.3390/ma12111824

**Published:** 2019-06-05

**Authors:** Sandra Pina, Viviana P. Ribeiro, Catarina F. Marques, F. Raquel Maia, Tiago H. Silva, Rui L. Reis, J. Miguel Oliveira

**Affiliations:** 1 3B’s Research Group, I3Bs—Research Institute on Biomaterials, Biodegradables and Biomimetics, University of Minho, Headquarters of the European Institute of Excellence on Tissue Engineering and Regenerative Medicine, AvePark, Parque de Ciência e Tecnologia, Zona Industrial da Gandra, 4805-017 Barco, Guimarães, Portugal; viviana.ribeiro@i3bs.uminho.pt (V.P.R.); catarina.marques@i3bs.uminho.pt (C.F.M.); raquel.maia@i3bs.uminho.pt (F.R.M.); tiago.silva@i3bs.uminho.pt (T.H.S.); rgreis@i3bs.uminho.pt (R.L.R.); miguel.oliveira@i3bs.uminho.pt (J.M.O.); 2 ICVS/3B’s—PT Government Associate Laboratory, 4805-017 Braga/Guimarães, Portugal; 3 The Discoveries Centre for Regenerative and Precision Medicine, Headquarters at University of Minho, Avepark, 4805-017 Barco, Guimarães, Portugal

**Keywords:** biomaterials, biopolymers, inorganic materials, scaffolds, hydrogels, porous structures, bioprinting, regenerative medicine, tissue engineering

## Abstract

During the past two decades, tissue engineering and the regenerative medicine field have invested in the regeneration and reconstruction of pathologically altered tissues, such as cartilage, bone, skin, heart valves, nerves and tendons, and many others. The 3D structured scaffolds and hydrogels alone or combined with bioactive molecules or genes and cells are able to guide the development of functional engineered tissues, and provide mechanical support during in vivo implantation. Naturally derived and synthetic polymers, bioresorbable inorganic materials, and respective hybrids, and decellularized tissue have been considered as scaffolding biomaterials, owing to their boosted structural, mechanical, and biological properties. A diversity of biomaterials, current treatment strategies, and emergent technologies used for 3D scaffolds and hydrogel processing, and the tissue-specific considerations for scaffolding for Tissue engineering (TE) purposes are herein highlighted and discussed in depth. The newest procedures focusing on the 3D behavior and multi-cellular interactions of native tissues for further use for in vitro model processing are also outlined. Completed and ongoing preclinical research trials for TE applications using scaffolds and hydrogels, challenges, and future prospects of research in the regenerative medicine field are also presented.

## 1. Introduction

Tissue engineering (TE) and regenerative medicine (TERM) have arisen as new biomedical fields that bring advanced approaches for damaged tissue regeneration and healing [1]. The field of TERM has significantly increased over the past decades, and its advances have involved a multitude of research, including biomaterials design and processing, surface characterization, and functionalization for improved cell-material interactions and imaging. Diverse approaches proposed include: (i) direct implantation into the defects of cells isolated from the patient [2]; (ii) bioactive molecules and growth factor delivery targeting tissue specificity [3]; (iii) cell-free scaffolding biomaterials [4]; and (iv) cell-laden scaffolding structures mimicking the natural extracellular matrix (ECM) of the tissues [5,6]. The latter ones are the most commonly used, which typically involve three-dimensional (3D) porous and hydrogels scaffolds, on which the cells grow and organize to form an ECM used in the regenerative process [5]. These 3D constructs deliver the physicochemical and mechanical maintenance for in vitro ECM formation, being slowly degraded, resorbed, or metabolized upon in vivo implantation [7,8]. The porosity, pore sizes, and interconnectivity of these structures hold a direct influence over their functionality. High porosity is important for allowing cell infiltration and ECM colonization, which is also directly influenced by pore size. Open and interconnected pores will benefit the growth, proliferation, and migration of the cells to an extent on ECM production. Additionally, the tissue vascularization and formation of new tissue may be faster [9]. On the other hand, the microporosity is also required for efficient cell adhesion and spreading, as well as for facilitating the initial mechanical strength between the scaffold and the tissue [10,11]. The degradation, biocompatibility, safety, stability, and cost-efficiency are also important considerations for clinical scenarios [12,13]. 

A broad variety of naturally derived and synthetic-based polymers have been applied for scaffold processing. The natural polymers have been showing biological properties that better fit to the regular microenvironment of tissues, promoting desirable cellular responses, biocompatibility, and degradability [14]. More recently, materials derived from decellularized ECM (dECM) have been widely explored in TERM. In fact, dECM preserves the native tissue composition, not only in terms of structural proteins as collagen, but also preserves growth factors and cytokines, which can improve cell growth and viability, and tissue repair and remodeling [15]. Further, dECM has been obtained by means of employing different processing methodologies and from a diversity of tissues, such as bone, cartilage, meniscus, tendons, skin and adipose tissue, urinary bladder, small intestinal submucosa, liver, and brain [16,17,18,19,20,21]. On the other hand, the lack of mechanical properties of those biomaterials can be overcome by means of using synthetic-based polymers or combining them with inorganic and ceramic materials to form composite structures with superior strength, osteoconductivity, and bioresorbability [22,23,24]. Using synthetic polymers can also improve the chemical stability and the micro and nano-structural features of the scaffolds, which positively affect the cell adhesion, spreading, growth, and ECM infiltration [25]. Thus, depending on the TERM strategy, different biomaterials and processing technologies should be considered in order to optimize the scaffold’s performance in terms of surface morphology and internal configuration. The most promising technologies proposed for scaffold processing include, among others, solvent casting with particulate leaching [26], freeze-drying [27], gas foaming [28], fiber bonding and electrospinning [29], phase separation [30], and more advanced technologies, such as 3D printing methodologies [31,32,33]. All of them have a great impact on mimicking human tissues for regeneration when combating chronic and degenerative diseases [34]. Nowadays, the field of TE has been revolutionized by the application of such technologies for developing bio-inspired models of complex tissue diseases for novel therapeutic drug screening and specific biomarker identification in patient-specific theranostic approaches [35]. Several studies have focused on the 3D character and multi-cellular interactions of native tissues, envisioning these 3D technologies as ideal for TERM in vitro model processing. The superior complexity and hierarchy of 3D engineered models have proved to better mimic the natural ECM of damaged tissues, simulating interactions between healthy–unhealthy cell types and the influence of the physical microstructure and mechanical properties of the native tissues [36]. Thus, the biomaterials, approaches, and emerging technologies applied for 3D scaffolds and processing of hydrogel matrices according to the final TERM application and native tissue complexity are herein presented. The multifunctional scaffolds with more complex biological functions and their usefulness for different TERM strategies are also explored. Clinical trials involving 3D scaffolds and hydrogel matrices, challenges, and future prospects of research in the TERM field are also underlined.

## 2. Biomaterials for Tissue Engineering and Regenerative Medicine

Current strategies for TERM involve the use of a wide pallet of materials, consisting of natural and synthetic polymers (e.g., proteins, polysaccharides glycosaminoglycans, poly-glycolic acid (PLG), polyl-actic acid (PLA), poly-ε-caprolactone (PCL), etc.), inorganic biomaterials, which include metals (e.g., titanium and its alloys, etc.) and ceramics (e.g., alumina, zirconia, CaPs, calcium phosphate cements (CPCs), etc.), and their hybrid combinations. Polymers have great stiffness and advantages are added to the natural polymers, namely from their similarity with the ECM, specific degradation owing to the susceptibility of the enzyme action, and improved recognition by the living body. Inorganic biomaterials are recognized for their biocompatibility, osteoconductivity and bioresorbability. The most promising polymers and inorganic biomaterials, as well as their hybrids, are described as follows.

### 2.1. Natural and Synthetic Polymers

Natural and synthetic polymeric materials are popular for engineering and regenerating hard and soft tissues due to their vast diversity of properties, such as biodegradation, mechanical properties, high porosity and surface-to-volume ratio, as well as small pore size [22,23,37]. Multiple applications for different type of polymers have been exploited in the current market for bone, cartilage, skin, wound healing vascular grafts, and tracheal splints [38,39].

**Figure 1:**
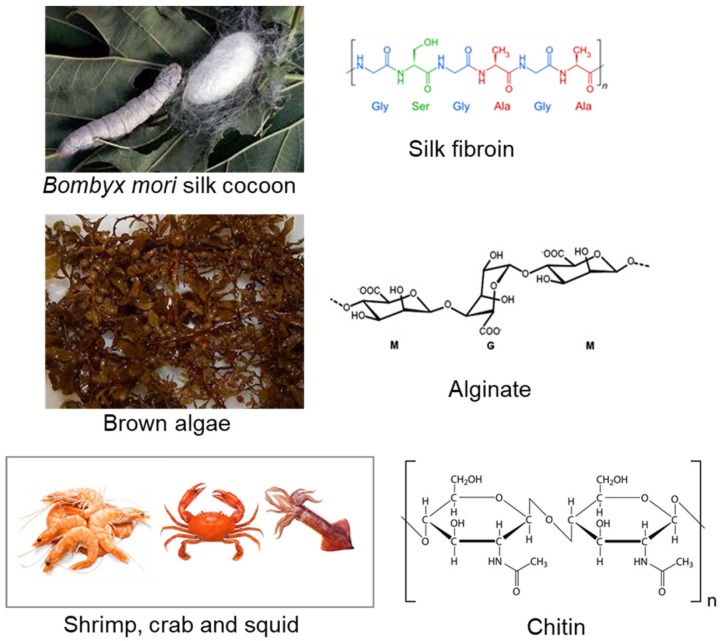
Some biopolymers derived from renewable resources and respective chemical structures: silk fibroin, alginate, and chitin.

Natural polymers obtained from renewable resources, such as algae, plant, animal, and microorganisms, are similar to biological macromolecules, and easily recognized by the environment (Figure 1) [40]. Owing to their similarity with the ECM, natural polymers, also known as biopolymers, may also elude chronic inflammation toxicity or immunological reactions, frequently noticed with synthetic polymers. Therefore, these types of polymers are crucial for designing therapeutic systems to be used as bioactive compounds and drug delivery systems for disease treatment, or even to bioengineer functional tissues. Biopolymers that have been clinically used for implant fabrication include proteins (e.g., silk fibroin, collagen, gelatin, keratin, fibrinogen, elastin, and actin), polysaccharides (e.g., chitosan, chitin, alginate, gellan gum, and derivatives), and glycosaminoglycans (e.g., hyaluronic acid) [40]. Structural proteins, such as elastin, fibrin, silk, and albumin, have been applied as sutures for scaffolds fabrication and as drug delivery systems [41,42].

Synthetic polymers, on the other hand, have excellent processing characteristics in terms of their molecular weight, degradation, and mechanical properties, with the advantage of having tailored property profiles for specific applications [43]. Hydrolytically degradable polymers are mostly chosen as implants due to their minimal site and patient-to-patient variations when compared to enzymatically degradable polymers [44]. However, many of these polymers present an immune response or toxicity, particularly when combined with certain polymers and not being capable of being incorporated with host tissues [45]. A strategy is to develop hybrid materials by combining them with natural polymers to improve hydrophilicity, cell attachment, and biodegradability. The most-used synthetic polymers in TE are polyglycolide (or poly glycol acid (PGA)), polylactide (or PLA), poly-lactide-co-glycolide (PLGA), poly-(D,L-lactic acid) (PDLLA), poly-ethylene-glycol (PEG), and PCL. These polymers can be self-reinforced to enhance their mechanical strength [46].

### 2.2. Inorganic Biomaterials

**Figure 2:**
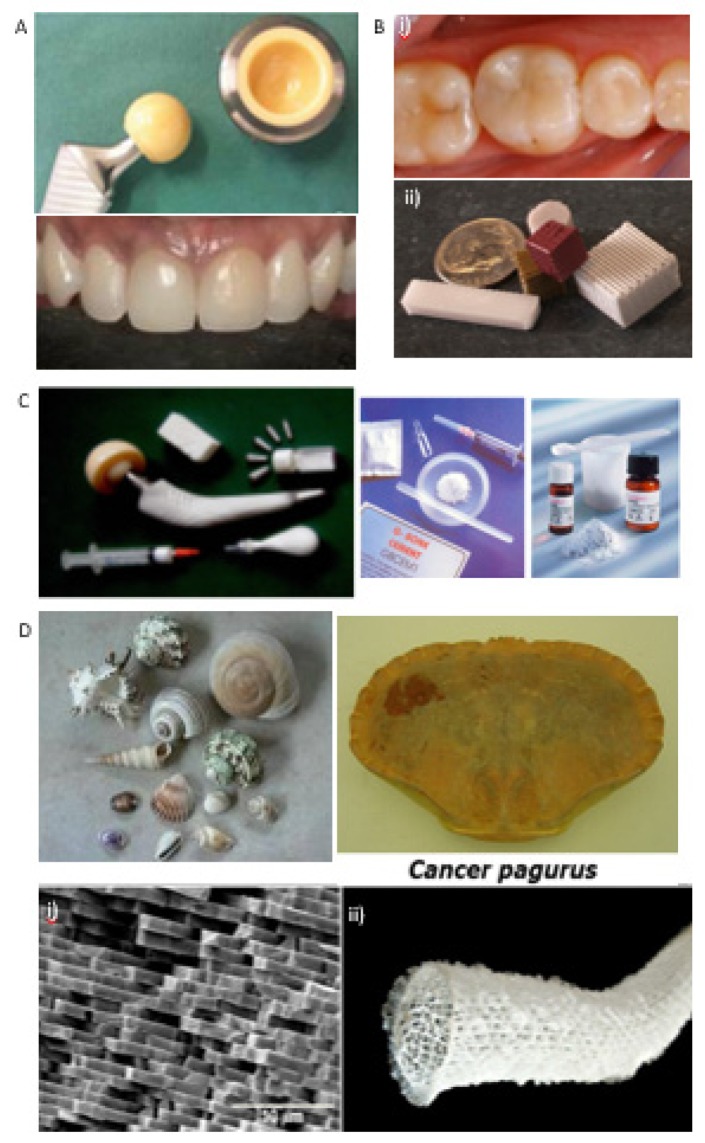
(**A**) Alumina/zirconia bioceramics for hip joint prosthesis and dentistry [67,68]; (**B**) (i) Bioactive glass-ceramics for dental applications and (ii) robocast glass scaffolds produced by scientists at Missouri University of Science and Technology [69,70]; (**C**) CaPs-based bone graft materials, such as porous blocks, powders and granules, hydroxyapatite (HAp) coating on a femoral metal stem, and self-setting CPC pastes that can be injected into the bone defect. Adapted from previous studies [71,72] with permission. (**D**) Images of marine organisms, namely shells, corals, sponges, and nacres, and microstructure image of the nacre structure, evidencing the plate-like aragonite crystals (**i**) and glass sponge (**ii**). Reprinted from a previous study [50] with permission.

An assortment of natural and synthetic inorganic biomaterials (metallic and ceramics) with particular compositions, microstructures, and long-term reproducibility have been proposed to repair or substitute diseased and damaged parts of the musculoskeletal system and periodontal anomalies (Figure 2). These types of biomaterials have been established for orthopedic load-bearing coatings (hip acetabular cups), bone grafting and cements, and dental restorations [47]. Metallic biomaterials (e.g., titanium and its alloys) possess high strength, low modulus of elasticity, and low density, while ceramics biomaterials, also known as bioceramics (e.g., alumina, zirconia, CaPs, calcium phosphate cements (CPCs), and silicates), are considered for their biocompatibility, osteoconductivity, and osteogenic capacity (Figure 2A–C) [48,49]. 

Inorganic biomaterials can be classified as bioinert, bioactive, or bioresorbable depending on their ability to bond directly with native tissues once implanted. Bioinert materials (e.g., alumina, zirconia, titanium, and its alloys) have no interaction with their adjacent tissue after implantation, typically being applied as structural-support implants, such as bone devices and femoral heads. On the other hand, bioactive materials (e.g., bioglasses and glass-ceramics) bond directly with living tissues, and have been applied to fill small bone defects and periodontal irregularities. Bioresorbable materials (e.g., CaPs, CPCs, and calcium carbonates or calcium silicates) gradually absorbed in vivo and are replaced by bone over time. 

Naturally-derived inorganic biomaterials from marine shells, corals, sponges, nacres, and animal (fish and chicken) bones offer an abundant source of calcium compounds (e.g., calcium carbonate and calcium phosphate) for TERM applications (Figure 2D) [50]. Coral-derived materials have been used as raw materials to obtain CaPs-based biomaterials for bone tissue repair and regeneration, owing to their microstructural and mechanical properties. Our group has been involved in the production of porous bioceramics using a variety of red algae (e.g., *Coralline officinallis*) [51,52]. This process involves a thermal and chemical treatment to convert calcium carbonate skeletons of *C. officinallis* particulates into CaPs with hydroxyapatite (HAp) nanocrystallites, while keeping the natural microstructure of the red algae [51].

Synthetic inorganic biomaterials, such as alumina and zirconia, bioactive glasses and glass-ceramics, and CaPs-based materials (e.g., sintered, coatings and cement pastes), are the ones commonly applied in TERM [53,54]. These biomaterials can be obtained by numerous methods (e.g., aqueous precipitation, hydrolysis, sol-gel synthesis, hydrothermal synthesis, mechanochemical synthesis, microwave processing, and spray drying), resulting in materials with increased crystal size and morphology [55,56,57]. Among them, the wet precipitation method offers an advantage on the material synthesis, which involves a precise control of the pH, temperature, particle morphologies, and the presence of additives [58].

A number of studies are dedicated to functionalizing bioactive inorganic materials by doping them with ionic elements (e.g., strontium, zinc, magnesium, manganese, silicon) that are slowly released during bone resorption, and therefore can boost biocompatibility and the mechanical strength of the implants [59,60,61,62,63,64]. Moreover, these minerals afford physicochemical modifications, thus accelerating bone formation and resorption in vivo [65,66].

### 2.3. Organic-Inorganic Hybrid Biomaterials

Hybrid biomaterials formed by combining organic and inorganic compounds result in multifunctional materials with tailored mechanical, thermal, and structural stability properties [73]. Concerning the fabrication of composite scaffolds, it is essential above all to attain a good compatibility between the phases and maintain the porous structure and the mechanical strength of the scaffolds [74,75]. Furthermore, nanostructured hybrids have also been preferred due to the nanosized features of the fillers, thus enhancing the bonding capacity of the tissue to the organic matrices that the individual materials cannot accomplish [76]. The nanoparticles have large surface areas when compared to the micro-sized fillers, thus contributing to upgraded mechanical properties, while retaining the biocompatibility and osteoconductivity, cell adhesion, and proliferation of the fillers [77,78].

Many combinations of polymers and inorganic materials have been proposed to engineer different tissues with enhanced osteoconductivity and mechanical properties, including polymers of natural origins (collagen, gelatin, silk, chitosan, alginate, hyaluronic acid, and gellan gum), synthetic polymers (e.g., PEG, PLA, PGA, PLGA and PCL), and bioceramics, silicates, bioactive glasses, and carbon nanotubes [79,80,81,82,83,84,85,86].

## 3. Scaffolding Strategies for Tissue Engineering and Regeneration

Recently, the approaches used in TERM have mainly been committed to 3D porous scaffolds and hydrogels, resulting in mechanically stable structures with controlled degradation rates and porosity for the transport of gases, nutrients, and regulatory factors.

A number of traditional technological approaches categorized into the foam replica method, particulate-leaching, freeze drying, gas foaming, and phase separation, have been applied for scaffold production, showing inexpensive and optimized physicochemical property structures. Lately, advanced manufacturing (e.g., 3D printing and robocasting), supercritical fluid technology, and microfluidics have emerged to produce complex structures for defective tissue regeneration, with boosted porosity, structural and mechanical properties, and cellular adhesion, providing several advantages over the conventional ones. 

A description of 3D porous scaffolds and hydrogel strategies is provided below.

### 3.1. 3D Porous Scaffolds

With the increasing need for advanced therapeutics for TERM, 3D scaffolds arise as porous matrices capable of providing a proper microenvironment for such purposes. The scaffolds should allow: (i) the transport of the nutrients needed to the cell attachment, proliferation, and differentiation; (ii) stimulation of cell-biomaterial attachment, growth, and migration; (iii) mechanical support; and (iv) a controlled degradation rate with no toxicity or inflammation risk to the cells [87].

As mentioned previously, different technologies and biomaterials have been applied in order to fabricate porous scaffolds with organized porosity and pore sizes, such as foam replicas, freeze-drying, phase separation, particulate-leaching, gas foaming, photolithography, microfluidics, supercritical fluid technology, stereolithography, robocasting, and 3D printing and bioprinting [81,88,89,90,91,92,93,94,95,96,97,98,99].

#### 3.1.1. Natural 3D Porous Scaffolds

**Figure 3:**
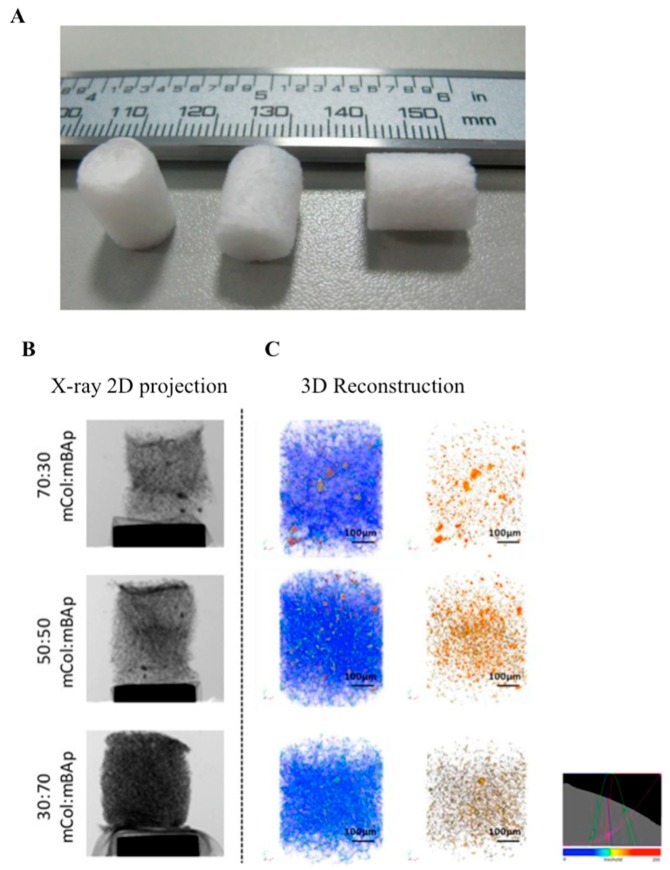
(**A**) Scaffolds of marine collagen: marine biopatite particles composite. Representative images of 12.5% 1-[3-(dimethylamino)propyl]-3-ethylcarbodiimide hydrochloride/N-Hydroxysuccinimide (EDC/NHS) crosslinked scaffolds obtained by microcomputed tomography (micro-CT). (**B**) X-ray 2D projection and respective (**C**) 3D reconstruction of acquired structures, in which the first column shows a reconstruction of both polymeric and ceramic phases, and the second column shows only the ceramic phase. Homogeneous distribution of the materials is observed, according to a color scale: blue = soft material (marine collagen); brown = hard material (marine biopatite particles). Adapted from a previous study [105] with permission.

Over the past years, our group has been involved in the fabrication of 3D porous scaffolds for hard TE applications, mainly using materials of natural origin [53,100,101,102,103,104]. In particular, the use of marine resources are an alternative to extract bioactive compounds, with which resources are isolated from by-products at low cost, thus creating value from products that are considered waste for the fish transformation industry. In a study by Diogo et al. [105], the fabrication of 3D scaffolds is reported using collagen from shark skin (*Prionace glauca*) combined with CaPs obtained from the teeth of two different shark species (*Prionace glauca* and *Isurus oxyrinchus*) through freeze-drying technique (Figure 3A). The produced scaffolds showed a homogeneous distribution of apatite particles throughout the collagen matrix able to support the attachment and proliferation of osteoblast-like cells (Figure 3B,C) [105].

**Figure 4:**
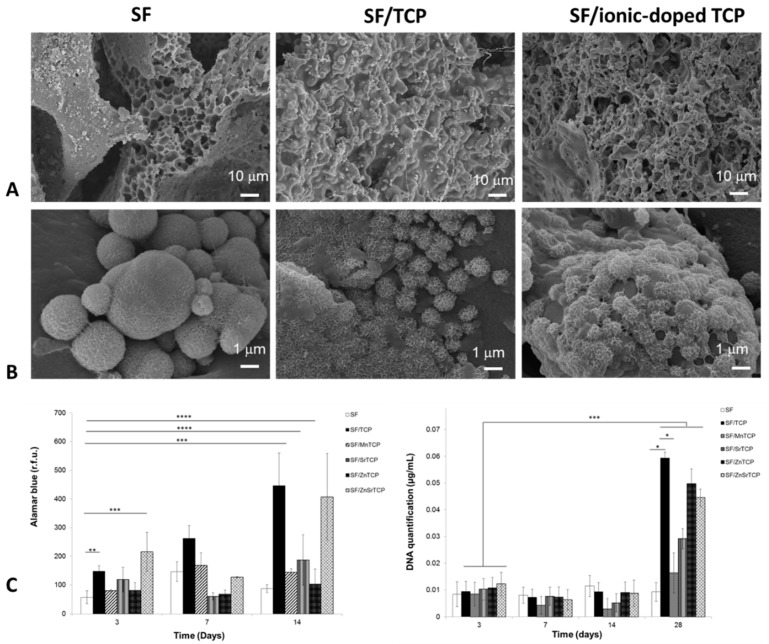
Scanning electron micrographs of silk fibroin (SF), SF/tricalcium phosphate (TCP), and SF/ionic-doped TCP scaffolds before (**A**) and after 15 days of mineralization (**B**). Viability and proliferation of the scaffolds seeded with human adipose-derived stem cells (hASCs): Alamar blue assay of hASCs cultured for 14 days (left), and DNA quantification at different time points (right) (**C**); *significant differences compared with SF and SF/Mn-doped TCP (MnTCP) and with SF/MnTCP and SF/Sr-doped TCP (SrTCP) (p < 0.05); **significant differences compared between SF and SF/TCP (p < 0.005); ***significant differences compared between SF at 3 d and the different compositions at 28 d (p < 0.0005); ****significant differences compared between with SF and SF/SrTCP and SF/ZnTCP (p < 0.0001). Adapted from a previous study [64] with permission.

In another study from our group, the development of biofunctional scaffolds was reported using a natural biopolymer containing silk fibroin (SF) and β-tricalcium phosphate (β-TCP) and incorporating strontium, zinc, and manganese via salt-leaching and a freeze-drying technique [64]. The scaffolds revealed highly interconnected macroporosity of 500 μm, and a microporous structure with a size range of 1–10 μm (Figure 4A). The scaffolds presented biomineralized globule-like structures of apatite crystals and porous spherulite-like structures with the incorporation of the ceramic part into the silk upon immersion in simulated body fluid for 15 days (Figure 4B). Remarkably, in vitro assays conducted with these biomaterials and human adipose-derived stem cells (hASCs) have shown different responses in terms of cell proliferation and differentiation when varying the doping elements in the scaffolds (Figure 4C). The presence of Zn led to improved cell proliferation, while the Sr- and Mn-doped scaffolds presented higher osteogenic potential, as demonstrated by DNA quantification and alkaline phosphatase (ALP) activity, respectively. The combination of Sr with Zn led to a significant influence on cell proliferation and osteogenesis in comparison to the single ions. Several studies have been reported using dECM-based scaffolds for TERM [17,18,19,106,107]. In fact, those types of scaffolds confer an ideal microenvironment, with instructive biological molecules and reduced immuno responses [108]. For example, Zhang et al. [17] prepared dECM from swine menisci together with gelatin/chitosan composite scaffolds, with enhanced elastic modulus and non-cytotoxicity properties for meniscus TE. The dECM-based scaffolds improved rat bone marrow stem cells (BMSC) proliferation when compared with scaffolds without dECM. In another study, Parmaksiz et al. [19] developed a multilayer scaffold of decellularized bovine small intestinal submucosa (bSIS) layers, together with HAp microparticles and PCL, with potential for bone TE. For that, bSIS layers were stacked with PCL solutions that acted as a glue, in order to improve the mechanical properties. Then, the multilayered PCL/bSIS scaffold was uniformly composited with ~30 μm HAp microparticles in the structure. In vitro studies have shown that rat BMSCs proliferated and differentiated along the osteoblastic lineage on the scaffolds within 21 days. Furthermore, the cell-laden scaffold revealed a maximum strength after 21 days of culture, close to the values of the cell-free multilayered scaffolds in wet conditions.

#### 3.1.2. 3D Printed Scaffolds

The development of TE scaffolding for soft-to-hard tissue regeneration by additive manufacturing (AM) has been widely reported [109,110]. Among the available AM techniques, robocasting (also called direct-write assembly) is a versatile technique that allows the production of scaffolds with predefined morphologies and structures, capable of fully supporting their own weight during assembly, allowing precise control of pore size, shape, and alignment [111,112,113]. Miranda et al. [114] optimized the morphological properties of TCP powders by reducing the particle size and increasing the specific surface area for robocasting use. The β-TCP scaffolds were printed with various geometries in the range of 10–20 mm/s. Moreover, the compressive strength of the scaffolds achieved was 10–20 MPa, similar to the corresponding values for the cancellous bone (7–10 MPa). Additionally, Heo et al. [115] produced HAp/PCL composite scaffolds through robocasting technique with a well interconnected macroporosity yielding a final porosity of 73% and a pore size of 500 μm. The compressive modulus of the micro-HAp/PCL and nano-HAp/PCL scaffolds obtained was 1.3 and 3.2 MPa, respectively. The more hydrophilic surface of nano-HA/PCL, which resulted from the higher surface area of nano-size HAp, could promote better cell attachment and proliferation compared with micro-HAp/PCL. Martinez-Vazquez et al. [116] reported that the incorporation of PCL or PLA into β-TCP porous scaffolds, fabricated by robocasting, increased the compressive strength of the scaffolds. More recently, Marques et al. [117] studied pure and Sr- and Ag-doped biphasic CaPs scaffolds, obtained via robocasting, for bone tissue regeneration. The scaffolds showed different pore sizes with compressive strengths comparable to or even higher than that of cancellous bone. Moreover, the presence of Sr and Ag improved the mechanical strength and cell proliferation, and granted good antimicrobial activity against *Staphylococcus aureus* and *Escherichia coli*.

**Figure 5:**
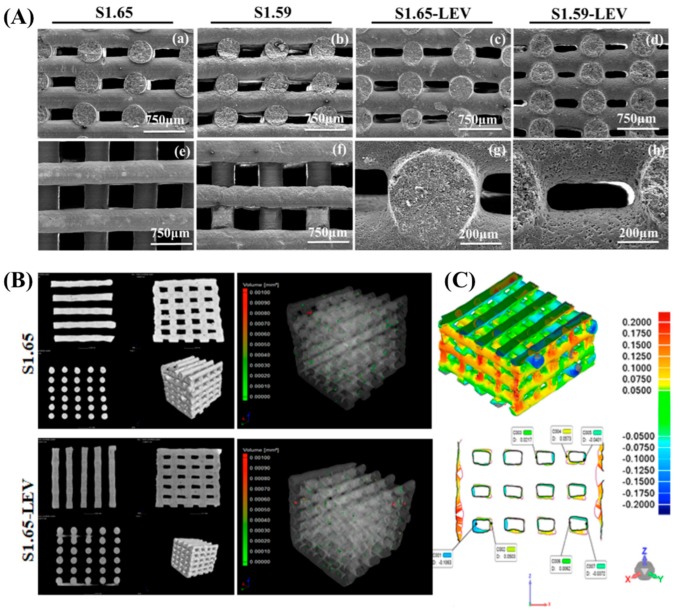
(**A**) Scanning electron micrographs showing morphological aspects of S1.65 (Ca/P = 1.65) and S1.59 (Ca/P = 1.59) scaffolds with and without levofloxacin: (**a**–**d**) lateral views; (**e**,**f**) top-views; (**g**) filament detail, (**h**) pore detail; (**B**) 2D (top plane and cross-section views) and 3D images of S1.65 scaffolds with and without drug (levofloxacin) obtained through Metrology CT and 3D reconstruction of the scaffolds mapped with color-coded for the internal porosity of filaments. (**C**) The 3D and 2D views of overlapped S1.65 and S1.65- levofloxacin (LEV) mapped with color-coding. Adapted from a previous study [119] with permission.

The incorporation of biomolecules in the scaffolds, such as growth factors, antibiotic, or anti-inflammatory drugs aimed at the acceleration of local bone healing, is currently under extensive research [118]. The robocasting technique is able to use a broad range of materials for the manufacture of scaffolds incorporating several biomolecules. Marques et al. [119] studied the processing conditions to obtain sintering-free composite scaffolds through robocasting (Figure 5), constituted by biphasic CaP (with Ca/P ratio of 1.65 and 1.59), chitosan, and levofloxacin (LEV) in the absence of processing additives (dispersant and binders). After robotic deposition, the scaffolds maintained the shape and no filament collapsing could be observed (Figure 5A,B). However, the overlapping of scaffolds, with and without antibiotics, shows that they could not be totally superimposed, because the LEV modified the viscoelastic behavior of the inks (Figure 5C). The LEV-loaded scaffolds exhibited an early and fast drug release, but also presented bacteria growth inhibition ability, proving that the antibiotic was not degraded during the fabrication process. Furthermore, its bactericidal effectiveness was preserved, which opens a new path for local bone regeneration and infection treatments, since a more direct administration of a drug might be a better solution than the conventional treatment strategies. With the same purpose of including relevant biomolecules in the scaffolds, bioprinted scaffolds coated with dECM were developed [107,120]. Wu et al. [107] prepared calcium silicate (CS) and PCL scaffolds and then cultured an osteoblastic cell line (MG63) on top of the scaffolds in order to produce a relevant ECM coating for bone TE. Upon removal of the cellular content, human Wharton’s Jelly mesenchymal stem cells (WJMSCs) were seeded on the scaffolds. In vivo studies using a rat critical defect were then performed. In turn, Kim et al. [120] developed PCL/β-TCP scaffolds and then immersed the scaffolds in a porcine bone dECM solution. After lyophilization, pre-osteoblastic cells (MC3T3-E1 cell line) were cultured onto the scaffolds, and in vivo studies were evaluated in a rabbit critical calvarial defect. In both studies, the printed scaffolds coated with dECM have been shown to enhance osteogenic differentiation in vitro, and the implantation of the scaffolds showed new bone formation, which validate the use of dECM in the improvement of scaffolds for bone TE. 

Recently, the 3D bioprinting technique has been commonly used in TERM. This technique has the advantage of allowing high freedom for cell and biomolecule positioning in diverse biomaterials with predefined designs and geometries [121]. Alginate is one of the most used biopolymer for 3D cell printing because it forms a stable hydrogel in the presence of divalent cations (e.g., Ca^2+^ or Ba^2+^) by ionic crosslinking [121,122]. However, alginate-based bioinks have some disadvantages, namely their biological activity, since they do not provide mammalian cell-adhesive ligands [123].This fact can be overcome by modifying the alginate surface with peptides, such as arginine-glycine-aspartate (RGD), to provide molecule binding sites for cell adhesion [124], for example by blending it with gelatin, which also allows the viscosity of the hydrogel to be altered to satisfy extrusion and printing criteria [125]. Another study investigated 3D bioprinting scaffolds for cartilage tissue by combining collagen type I or agarose (AG) with sodium alginate (SA) incorporated with chondrocytes [126]. The results showed that the addition of collagen or AG had a little impact on the gelling behaviour and can improve the mechanical strength when compared to SA alone. Furthermore, the presence of collagen facilitated cell adhesion, accelerated cell proliferation, and enhanced the expression of the cartilage specific genes, namely *Acan*, *Sox9*, and *Col2a1* [126]. Also, Lee et al. [127] produced cell-laden collagen-based scaffolds using a dispensing system with tannic acid as a crosslinker. The cellular activities using MC3T3-E1 cells and the tannic acid crosslinking process revealed their capability of supporting high cell viability with reasonable biocompatibility of the developed scaffolds. In another study, Kim et al. [128] developed a new strategy to fabricate a α-TCP/collagen cell-laden scaffold with pre-osteoblasts MC3T3-E1 cells for bone tissue repair. The results showed that the α-TCP/collagen scaffolds had significantly higher cellular activities compared with those of the controls, including metabolic activity and mineralization, as well as good mechanical properties.

Recent cellular and acellular reported studies using different scaffold strategies for TE purposes are summarized in Table 1.

### 3.2. Hydrogel-Based Scaffolds

Hydrogels are of particular interest for TE applications due to the distinctive properties of matrices formed through 3D networks. Particularly, hydrogel-based systems are highly hydrated structures that result from crosslinking reactions of polymers with hydrophilic natures that resemble the natural ECM of tissues [149]. Such properties ensure a suitable microenvironment for cells to grow, drug incorporation, and controlled release of biologically active agents. The elastic behavior and swelling capability of hydrogels makes them desired for injectable purposes and bioprinting applications, which is an emerging technology for the 3D fabrication of structures used for the construction of complex functional tissues and artificial organs, from nano- to macro-scales [150]. This innovative technology revolutionized the TERM field, not only because of the complexity of the biocompatible matrices, but also because of the opportunity to integrate cells and supporting components into the complex 3D functional architectures produced for transplantation. Compared with non-biological 3D printing, technical challenges related to the sensitivity of living cells to the shear stress during the bioprinting process can be found [151], which requires the integration of knowledge in the fields of engineering, biomaterials science, cell biology, and physics. Bioprinting techniques have already been proposed for the fabrication of 3D hydrogel-based structures, envisioning several tissue transplantations or substitutions, including skin [152], bone [153], vascular grafts [154], intervertebral disc (IVD) [102], meniscus, and cartilage [155]. More recently, the development of high-throughput in vitro platforms of healthy and diseased tissues of the human body came to address the TE field to a different level of precision medicine [156], and the 3D bioprinted hydrogels emerged as highly precise biomimetic matrices [157]. Apart from their use as aqueous-based systems for cell encapsulation [158], as injectable fillers [159], or in bioprinting technologies [160], different processing methodologies can be applied for structuring hydrogels into highly porous and composite matrices with superior mechanical properties, including solvent casting and particulate leaching, freeze-drying, phase separation, gas foaming, electroforming, and polymer blending [161,162,163]. These technologies have been proposed using different natural- and synthetic-based polymers, whose selection criteria depends on their chemistry, molecular weight, solubility, and hydrophilicity or hydrophobicity [164]. As aforementioned, the polymers of natural origin are in most cases an attractive option, mainly due to their similarities to the ECM and suitable biological performance [40]. However, their chemical versatility also brings molecular instability that can compromise hydrogel stability, degradability, and reproducibility [165]. On the other hand, the synthetic polymers are of controlled reproducibility and usually present superior mechanical properties and slow biological degradation, making them ideal for hard tissue applications or as indirect scaffolding strategies, serving as the structural basis for natural-origin hydrogels [166].

#### 3.2.1. Injectable Hydrogels

Injectable hydrogels are highly attractive, especially as fillers of soft and hard tissues, promoting a good physical integration into the defect site and possibly avoiding open surgeries with hard recovery of the patients. The high water content of these hydrogels make them adjustable and easy to manipulate for the delivery of cells and growth factors. Usually, the hydrogel precursors are injected into the wound site in a solution-to-gelation transition (sol-gel) due to physical or chemical stimuli and crosslinking reactions [167]. The most common physical crosslinking methods for in situ hydrogelation reactions take place by the physical association between polymeric chains or nanoparticles, and include thermal gelation, ionic interactions, physical self-assembly, or photopolymerization [168,169,170]. The formation of chemically-induced hydrogels occurs via covalent bonds between polymeric chains promoted by agents such as glutaraldehyde or genipin and enzymes [171,172]. The physical methods of crosslinking, such as thermal gelation in physiological conditions, are easy to process and do not involve limitations of injection depth, as in the case of photopolymerization methods [173]. These crosslinking mechanisms can be even harder to control when applied in natural polymers, such as collagen or fibrin, which limits the final structural properties of the produced hydrogels. Kim et al. [174] reported chitosan/β-glycerophosphate (Ch/β-GP) thermo-sensitive hydrogels formed to deliver ellagic acid in cancer treatment. The heat-induced hydrogels were formed at body temperature but the final pH of the Ch/β-GP solution affected the gelation temperature, time, and biocompatibility within the gels. The suitability of chitosan/β-glycerophosphate to produce injectable thermosensitive and pH-dependent hydrogels was also investigated in combination with starch, showing that its addition to the chitosan/β-glycerophosphate solution did not alter the transition temperature and allowed the heating induced hydrogelation for applications in minimally invasive injectable systems [175]. Furthermore, thermal gelation is the main crosslinking method for obtaining dECM hydrogels. An example is the study of Alom et al. [176] that developed a decellularized and demineralized bovine bone ECM (bECM), and upon thermal induction, obtained a hydrogel suitable for bone regeneration. In fact, it was observed that Pluripotent myoblast C2C12 cell line and mouse primary calvarial cells (mPCs) cultured on top of bECM differentiated even in the absence of osteoinductive supplements.

**Figure 6:**
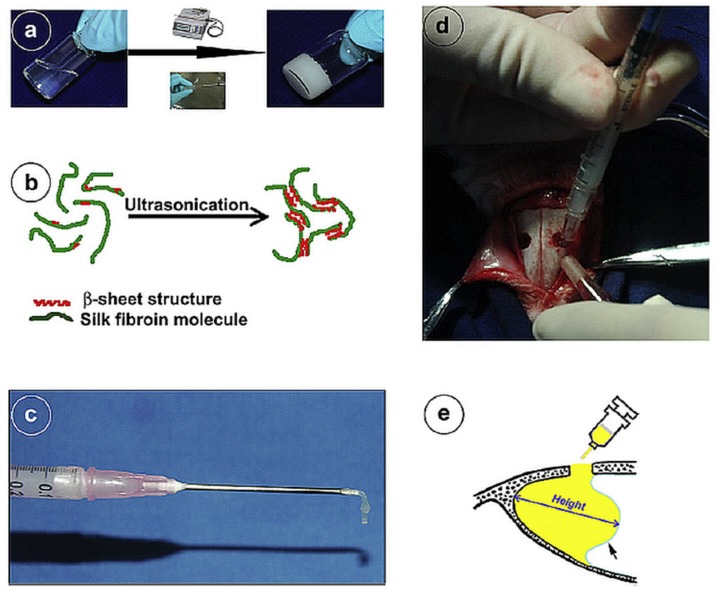
Sonication-induced silk fibroin hydrogel preparation. (**a**) Ultra-sonication procedure used for aqueous silk solution transformation into a solid silk hydrogel. (**b**) Schematic illustration of the mechanism of silk sol-gel transition due to physical crosslinking and β-sheet aggregate formation after ultra-sonication. (**c**) Injectable properties of the silk hydrogels. (**d**) Silk hydrogel being injected into rabbit maxillary sinus cavity. (**e**) Illustration of the elevated sinus in a sagittal plane being filled by the injected silk hydrogel. Reprinted from a previous study [179] with permission.

Injectable hydrogels were proposed by Park et al. [177] as cartilaginous fillers composed of methacrylated glycol chitosan and hyaluronic acid photo-crosslinked with a riboflavin photoinitiator under visible light. The authors showed that a minimum radiation time was needed to produce stable hydrogels for cell encapsulation and chondrocyte viability. However, superior irradiation times that improved the hydrogels’ mechanical properties for deep hydrogelation also compromised cell viability. Townsend et al. [178] pursued a photo-crosslinked method in order to develop a methacrylated decellularized cartilage hydrogel (MeSDCC) with HAp nanofibers (HAPnf), bioglass microparticles (BG), or rat BMSCs for calvarial bone regeneration. Despite the increase of the mechanical stiffness provided by the HAPnf and BG, the authors observed minimal bone regeneration in vivo for all conditions. The chemical methods used for producing hydrogels have been shown to offer controllable structural properties due to the covalent bonds between the polymeric chains, particularly due to the crosslinking density, which can be adjusted according to the polymer origin and tissue application [167]. Silk fibroin (SF) is a natural polymer proposed as an injectable filler of bone and cartilage tissues defects, due to its superior mechanical properties, biocompatibility, and in vivo degradation profile [179,180]. Different studies have shown that the sol-gel transition on SF hydrogelation can occur due to different physical and chemical methods, including mechanical agitation, ultra-sonication, thermal treatment, pH variations, organic solvents (methanol), ionic species (Ca^2+^), or blending with other polymers containing hydroxyl groups (alginate, chitosan, or hyaluronic acid) [179,181,182,183,184,185] that induce the protein conformation transition from random coil to β-sheet (β-sheet aggregates formation) [186] (Figure 6), or the crosslinking of fibroin molecules in the aqueous solution [187]. A different approach was recently proposed for SF hydrogel formation in random coil conformation, involving the enzymatic crosslinking of aqueous SF solutions promoted by the horseradish peroxidase (HRP)/hydrogen peroxide (H_2_O_2_) complex [188,189]. In this system, the hydrogelation process was conducted in physiological conditions and the formed hydrogels underwent a spontaneous conformation transition to β-sheet over time. They showed timely and thermally responsive gelation properties, with tunable mechanical properties and viscoelastic properties of injectable matrices. Moreover, the possibility of encapsulating cells allow their viability and proliferation in the amorphous state, suggesting their use as artificial in vitro models for 3D microenvironment of tissue disorders and tumours.

#### 3.2.2. 3D Printed Hydrogels

**Figure 7:**
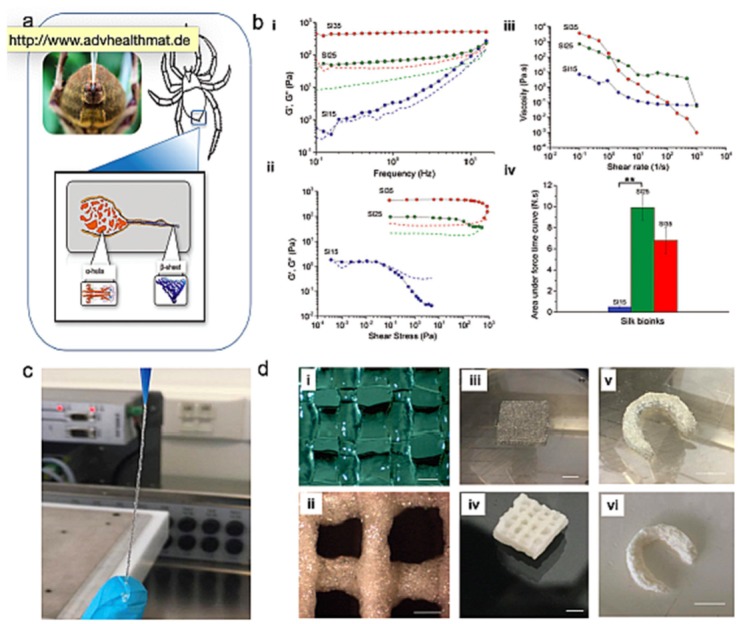
Silk fibroin bioinks and 3D printing of hydrogel-based scaffolds. (**a**) Schematic illustration of silk being extruded by a spider. (**b**) (**i**–**iii**) Rheological properties and (**iv**) adhesion measurements of the 3D printed hydrogels. (**c**) Extruded horseradish peroxidase (HRP)-crosslinked SF bioinks. (**d**) HRP-crosslinked SF scaffolds in the (**i**,**iii**,**v**) amorphous state and (**ii**,**iv**,**vi**) β-sheet crystalline conformation after freeze-drying and ethanol treatment. Reprinted from a previous study [192] with permission.

3D printed hydrogels are produced through computer-assisted technologies, allowing fabrication of engineered tissues or matrices with superior control over their shape and reproducibility, with controlled physical and mechanical properties, and different layers and gradients, allowing generation of more complex tissue-like 3D architectures [190]. 3D printing technologies applied in cell-free approaches are well standardized and have been proposed using different hydrogel-based systems for conventional TERM strategies. For instance, Li et al. [191] proposed 3D printed hydrogels as OC defect fillers using alginate and hyaluronic acid as photo-polymerized bioinks. The OC tissue was restored by reverse engineering using high-resolution 3D scanning to obtain digital models of sample defects and corresponding parts after regeneration. The information was translated to the 3D printer, which extruded the combined hydrogel filaments in order to achieve the precise shape of the OC defects. Thus, the combination of 3D digital technologies with 3D printing was suggested as a possible solution to treat complex skeletal lesions in patient-specific approaches. Also, Costa et al. [102] have proposed a reverse engineering strategy to fabricate 3D models of annulus fibrosus (AF) as the outer region of IVD. In this strategy, semi-automatic morphological segmentation from magnetic resonance was used to image the dataset of human IVD, and then HRP-crosslinked SF/elastin hydrogels were used as bioinks for the printing of AF substitutes. In this study, HRP-crosslinked SF hydrogels are proposed for the first time as fast-setting bioinks for 3D printing of hydrogels in the amorphous state [192]. Their properties were fine-tuned for specific uses, presenting good resolution, reproducibility, and reliability (Figure 7). Moreover, the structures presented excellent mechanical properties and memory-shape features after processing, exhibiting potential applications in patient-specific strategies. A fourth (4D) generation of printed hydrogels was proposed by Gladman et al. [193] thatprinted composite hydrogels encoded with localized and anisotropic swelling properties promoted by the alignment of cellulose fibrils pre-established in the printing settings. It was shown that this nature-inspired shaper-morphing system presented biocompatible and flexible bioink properties, opening the design of new stimuli-responsive architectures for TE and biomedical applications. 

**Figure 8:**
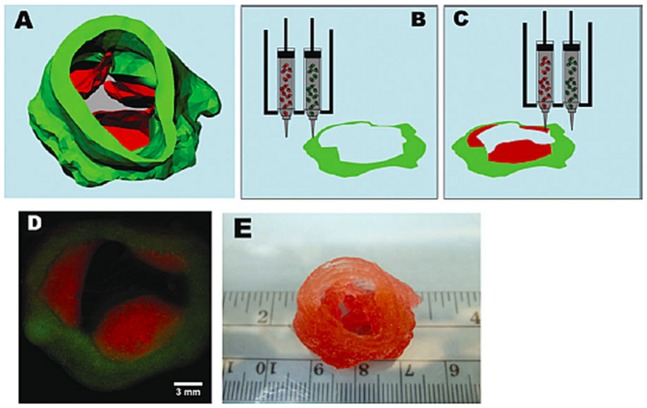
The 3D bioprinting of an aortic valve conduit. (**A**) The 3D reconstruction of an aortic valve model. The green color indicates valve root and the red color indicates valve leaflets. Schematic illustration of the 3D bioprinting process using alginate and gelatin as bioinks encapsulated with (**B**) sinus smooth muscle cells (SMCs) and (**C**) aortic valve leaflet interstitial cells (VIC) cells. (**D**) Fluorescent image of two 3D bioprinted layers representing an aortic valve conduit. (**E**) Macroscopic image of a 3D bioprinted aortic valve conduit. Reprinted from a previous study [196] with permission.

While the 3D printing technology is actually being applied for several TE and biomedical applications, it is a complex technology, since it involves not only computing and materials sciences, but also the interplay with other disciplines, such as cell-loading and developmental biological factors [31]. The interaction of multi-disciplinary technologies allows the development of more complex systems that better mimic the microarchitecture of tissues and organs. However, such complexity also creates a challenge in the design of functional 3D bioprinting. For example, the bioprinting parameters need to consider not only the heterogeneity of the polymeric materials (natural or synthetic) selected as bioinks, but also the influence of the cell-material dynamisms in the printing process. The shear-stress provoked during bioprinting not only affects the printing resolution but also cell viability and integrity [151]. Recent developments on creating spatial and temporal gradients within bioinks for regulating different cellular and molecular distributions along the hydrogels have also proved the increasing complexity in 3D bioprinting technologies [194]. For example, multiphase complex tissue structures of tendon-bone interface were obtained by creating multilayered gradients of encapsulated human mesenchymal stem cells (hMSCs) and growth factors (BMP-2 and TGF-β1) embedded in anisotropic 3D hydrogels [195]. The controlled deposition of two or more cell populations in co-culture systems using 3D bioprinting has been attracting much attention for the production of engineered tissues with superior biological, biochemical, and physical properties. Duan et al. [196] implemented a 3D bioprinting system for the direct incorporation of dual cell types, sinus smooth muscle cells (SMC), and aortic valve leaflet interstitial cells (VIC) encapsulated in alginate and gelatin hydrogels for cardiovascular tissue. Reverse engineering was applied using micro-CT imaging of heart valves for the direct recreation of anatomically accurate aortic valve conduits through 3D bioprinting (Figure 8).

In a different study, Gaebel et al. [197] applied a Laser-Induced-Forward-Transfer (LIFT) cell printing technique to prepare cardiac patches for cardiac regeneration. A Polyester Urethane Urea (PEUU) was used as bioink for umbilical vein endothelial cells (HUVECs) and hMSCs bioprinting as pre-defined cardiac patches. The authors showed that the LIFT-derived cell seeding pattern affected cell growth of co-cultured HUVECs and hMSCs that migrated and accelerated vessel formation in the simulated cardiac patches. An increase in capillary density was also observed after cardiac patch transplantation in the myocardium of infarct-induced rats. All of these systems were optimized in order to recreate heterogeneous tissue and organs by precisely co-printing multiple cell-loaded materials with 3D architecture. Kolesky et al. [198] implemented a new custom-designed 3D bioprinter with four independent printheads able to sustain four different bioinks. Heterogeneous constructs consisting of HUVECs, human neonatal dermal fibroblasts (HNDFs), and 10T1/2 mouse fibroblasts were printed with a perfusable microvasculature network using gelatin methacrylate (GelMA) as bulk matrix and cell carrier. Using this highly scalable platform, the vascular network and multiple cell types were programmed to be precisely placed within the ECM simulator, refining the multi possibilities of 3D reconstruction of complex tissues and organs using bioprinting technologies. 

In a different approach, the complex microstructure of the musculoskeletal system was mimicked using 3D integrated organ printing technology [199]. This system was used for processing and depositing four different components of an integrated muscle-tendon unit (MTU). Polyurethane (PU)-based hydrogels were used as bioinks and co-printed with C2C12 cells to develop the muscle region, and with NIH/3T3 cells for tendon development. The authors showed that the single construct was able to comprise the elastic properties of the muscle region and the stiffness of the tendon region only by using different cell types. The complex cell–matrix interactions were recaptured to form tissue constructs with region-specific biological and mechanical features. 

#### 3.2.3. Porous Hydrogels

**Figure 9:**
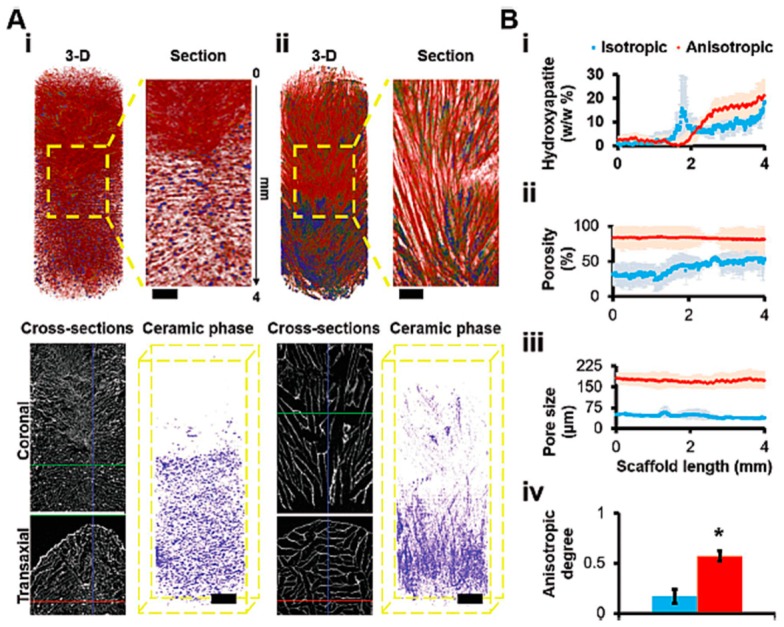
Structural characterization of gradient-induced 3D hydrogels. (**A**) The 3D reconstructions of: (**i**) random and (**ii**) linear porous architectures (isotropic and anisotropic). Red color represents methacrylated gelatin (GelMA)-methacrylated gellan gum (MAGG) blended polymers and blue color represents the hydroxyapatite (HAp) (Scale bar = 0.5 mm). Coronal and transaxial sections of the bilayered phased structures are represented, showing the continuous interface created throughout the matrices. The ceramic phase distribution is shown in purple inside the structure’s volume (Scale bar = 1 mm). (**B**) Quantification profiles of (**i**) HAp distribution, (**ii**) porosity percentage, and (**iii**) mean pore size traced for isotropic and anisotropic structures with linear and random pore distribution. (**iv**) Anisotropic degree assessed for each porous architecture, showing higher values on the linear porous anisotropic structures. Data of anisotropic degree was represented as mean ± SD, ranging from 0 (isotropic) to 1 (anisotropic) (*P*-value = 0.05, N = 3). Reprinted from a previous study [205] with permission.

As mentioned previously, the biocompatibility and structural similarities of hydrogels to the native ECM make them desirable for engineering different complex tissues. However, the big challenge remains to obtain precise control of certain hydrogel properties, such as porosity and mechanical properties. For certain tissues, especially those of the musculoskeletal system, a substantial amount of scaffold porosity is necessary to allow cell infiltration for ECM formation and secretion throughout the engineered tissues. Increased porosity and pore size can benefit the structure interconnectivity and the diffusion of nutrients and oxygen, especially in the absence of a pre-vascularized system, which is part of the microarchitectural composition of some tissues, such as cartilage, meniscus and IVD [166,200]. Another issue associated with hydrogels are their low mechanical properties and structural stability associated with the high-water-content that mimics the native ECM of tissues and allow cell encapsulation [201]. Thus, for some engineered tissues the possibility of structuring hydrogel-based matrices is a key role for regulating their microarchitecture and to improve many aspects of cell orientation, aggregation, and ECM function [202]. For example, in genipin crosslinked gelatin hydrogels with low porosity and pore size, the tendency of cells was to grow indiscriminately rather than produce and secrete ECM [203]. Consequently, the extent of ECM secretion was lower in these matrices as compared to that observed in gelatin hydrogels with larger pores, confirming that the porosity and pore interconnectivity greatly affect cell growth and penetration in the 3D structure of hydrogels. In a different study reported by our group [103], HRP-crosslinked SF hydrogels were structured using combined salt-leaching and freeze-drying methodologies for cartilage TE applications. The produced macro- and micro-porous SF hydrogels supported chondrogenic differentiation of human adipose-derived stem cells (hASCs) with a high degree of ECM formation and distribution within the porous matrices. The same matrices were further combined with macro- and micro-porous HRP-crosslinked SF hydrogels incorporating β-TCP particles and used as bone-like layers in an osteochondral (OC) TE approach [162]. The bilayered structures presented high porosity and homogeneous pore distribution, with mechanical properties suitable for bone-cartilage tissue applications. The co-culture of human osteoblasts and human articular chondrocytes on the respective layers of the bilayered structures showed that both cell types adhered, proliferated, and produced their respective ECM in the cartilage- and bone-like compartments. As previously mentioned, the same HRP-crosslinked SF hydrogels have been proposed as aqueous hydrogel systems for 3D printing applications, and possibly injectable purposes [189,192]. However, some stability drawbacks were detected during the printing process of the amorphous SF, namely the hydrogel’s mechanical resistance and stability in aqueous solution. Thus, through the salt-leaching processing or ethanol treatment applied to the HRP-crosslinked SF hydrogels, it was possible to induce a desired porosity and improve the hydrogel’s structural stability due to the protein folding and β-sheet formation [102,161,162]. A new methodology to produce SF-based hollow tubular conduits (TCs) was described by Carvalho et al. [204], using the HRP-crosslinked SF hydrogels processed through three different methods (freeze-drying, drying at 50 °C, permanent hydrated state), forming a crystalline β-sheet conformation after ethanol treatment. This approach allowed modulation of different characteristics of the final TCs, showing the hydrogel-based tubes with microstructures that ranged from nonporous to highly porous networks, which were selectively permeable to 4 kDa molecules but not to human skin fibroblasts. The porous conduits also presented high tensile properties and good resistance to the applied loads. The fabrication of gradient structures brings a particular interest in the TE field, not only for the development of high-throughput 3D bioprinted materials, but also for the creation of hydrogel-based structures with controlled porosity and pore size [196]. Following this strategy, Canadas et al. [205,206] proposed in different studies the fabrication of innovative 3D architectures with linear or random porosity produced using gradient distributions induced by freeze-drying processing, varying temperatures, and guided crosslinking. ECM-like networks of OC tissue [205] and neuronal organization [207] were made of a photo-crosslinkable blend of methacrylated gellan gum (MAGG) and gelatin (GelMA) hydrogels, showing the ability to form different isotropic and anisotropic structures with tunable pore sizes and porosities according to the desired application (Figure 9). The possibility of forming gradient distributions using HAp microparticles in combination with growth factors was also demonstrated, as well as the development of a heterotypic-like OC tissue with cell orientation guided by a dual chamber bioreactor [205]. It was shown that by using a control over polymeric matrices composition, crosslinking directional properties, and freezing gradient dynamics, it was possible to develop 3D hydrogel-based structures with top-down tunable properties in terms of macro- and micro-porosity, and capable of guiding cell orientation and ECM formation. These works opened new opportunities of developing more complex tissue models for new drug testing and patient-specific therapies in regenerative medicine.

The formation of bioactive polymer/inorganic hybrid hydrogels emerged as a new strategy for improving hydrogel mechanical stability, while porosity could also be increased [208]. Most of these strategies were developed for hard tissue applications, such as bone or OC complex, and include CaPs (HAp, α,β-TCP, or biphasic CaP) or bioactive glass or glass-ceramics incorporation within the polymer-based hydrogel matrix [162,207,208,209,210]. For example, Ma et al. [211] proposed biomimetic hybrid hydrogels consisting of collagen, Hap, and alendronate for bone TE applications. First, the anti-osteoporosis drug alendronate was conjugated with bioactive HAp particles, further incorporated within the hydrogel matrix formed from collagen, and using genipin as the crosslinker. The authors have showed that the hybrid hydrogels formed in physiological conditions exhibited remarkable mechanical properties, higher gel content, and lower swelling rations as compared to the non-hybrid hydrogels prepared exclusively from collagen. In a different study, injectable composites were proposed for bone regeneration applications by combining alginate as a hydrogel matrix with crystalline CaP powders as dispersed minerals or used as sources of calcium for alginate crosslinking [212]. The viscoelastic properties of hydrogels were tunable according to CaP content, while still maintaining their injectability to fill bone defects. The biocompatibility and viscoelastic properties of alginate-based hydrogel matrices combined with the osteoconductivity of CaP particles were beneficial for bone regeneration and showed promising results as minimally invasive bone-filler materials. Jiang et al. [213], designed stratified OC grafts with multi-tissue regions, based on an agarose hydrogel matrix integrating composite microspheres of PLGA and 45S5 bioactive glass (BG). The authors observed the ability of producing three distinct yet continuous regions of cartilage, calcified cartilage, and bone-like matrices by the incorporation of the PLGA-BG composite that promoted chondrocytes mineralization in the interface and the formation of a mineralized matrix in the osteoblast-cultured bone-like region. In addition, the PLGA-BG phase improved the mechanical properties of the multi-phased scaffolds, as compared to those prepared with PLGA alone and used as control.

In Table 2 some of the most recently reported studies are summarized using the aforementioned technologies and crosslinking methods to produce hydrogel-based matrices for TE purposes. 

## 4. Clinical Trials on Tissue Engineering/Regeneration

Human clinical trials or interventional studies are conducted to evaluate biomedical or behavior interventions, including new treatments, after approval of the health ethics committee. This process involves multiple stages of R&D studies before reaching the final stages of approval from the U.S. Food and Drug Administration (FDA). The FDA is a science-based agency in the US Public Health Service possessing legislative authority for premarket approval and post-market surveillance, and enforcement for a wide range of products in its regulatory preview. Research and development stages ensure the effectiveness and safety of new products and devices, which involve the production of medical grade scaffolds followed by animal testing under regulatory approved conditions. Over the last years, the research in TERM has resulted in few clinically approved therapeutics, but many more products are under development. Table 3 provides the completed and ongoing (with no reported results so far) preclinical research trials for TE applications using scaffolds and hydrogels in the last 5 years.

There are already some products with regulatory approval, such as: (i) bioceramics-based bone grafts substitutes, specifically CERAMENT™G, Bonalive (Vivoxid Ltd., Turku, Finland), NovoMax® (BioAlpha Inc., Bandar Baru Bangi, Malaysia), ChronOs (DePuySynthes, Raynham, MA, USA), Straumann® BoneCeramic™ (Basel, Switzerland), and Geistlich Bio-Oss® (Wolhusen, Switzerland); (ii) scaffolding materials for cartilage repair and regeneration, namely Cartilage Repair Device (Kensey Nash Corp., Exton, PA, USA); and (iii) biomaterials for OC defect regeneration, such as MaioRegen® (Finceramic, Faenza, Italy), TruFit^®^ (Smith and Nephew, Andover, MA, USA), and Cartilage Repair Device (Kensey Nash Corp., Exton, PA, USA). For small chondral and subchondral lesions treatment, there are also three off-the-shelf implants, namely ChondroMimetic® (TiGenix, Cambridge, UK), which is a collagen and CaP-bilayered scaffold, Agili-C^TM^ (Rizzoli Orthopedic Institute, Bologna, Italy), an aragonite-based cell-free implant, and Chondrofix^®^ (Zimmer Biomet, Warsaw) Allograft, which is a human decellularized hyaline cartilage and cancellous bone scaffold [220,221,222,223,224]. Marketed dECM scaffolds, harvested from several allogenic or xenogenic tissue sources, currently used in TERM are for the porcine small intestine (RestoreTM, DePuy Orthopedics; SurgiSIS®, Cook Medical, Bloomington, IN, USA) and liver (MIRODERM®; MIROMESH®; Miromatrix Medical Inc., Eden Prairie, MN, USA), human dermis (AlloDermTM, LifeCell Corp., Branchburg, NJ, USA), porcine urinary bladder (MatriStemTM, ACellr, Inc., Lafayette, IN, USA), bovine pericardium (PhotoFix TEM, CryoLife Inc., Kennesaw, GA, USA), and porcine heart valve (PrimaTM Plus, Edwards Lifesciences LLC, Synergraft®; CryoLife, Kennesaw, GA, USA) [225,226,227,228].

## 5. Concluding Remarks and Future Perspectives

There is a socioeconomic need to fully treat and replace damaged or non-functional tissues with pioneering approaches, designs, and technologies, converging on functional tissue reconstitution. TERM has appeared as a developing field based on materials engineering, biology, and medical knowledge struggling to produce alternative methods for the regeneration and repair of damaged tissues. Innovative strategies, such as the ones mentioned in this study, present out-of-the-box solutions for some of the current challenges in the TERM field, and may constitute foremost breakthroughs in the future. Such approaches will provide the production of bulk bioactive temporary implants with specific porosity and structure to contribute to the formation of new tissues for completing the medical tasks. The 3D scaffolds and hydrogel-based matrices are capable of meeting the challenges of personalized medicine, bringing effective treatments for an extensive assortment of pathologies. Hydrogel-based matrices have received a considerable interest for engineered tissue scaffolds owing to their structural similarities to natural ECM, as well as high water content, stiffness, and desirable structure for cellular proliferation. Furthermore, 3D bioprinted hydrogels arose as highly precise biomimetic matrices for the development of high-throughput in vitro models. Indeed, among the existing manufacturing technology, 3D bioprinting has been rapidly increasing with unlimited advantages of micro scale, high throughput, and cell deposition. Parallel to these advances, dECM has become popular in the TERM field owing to its ability to inherit the native ECM. In fact, besides the retention of the structure and biomolecules of the original tissues, dECM can be used for scaffolds, hydrogels, or even bioinks, alone or in combination with other materials, embracing different tissues. Nevertheless, there are still some issues, namely its low reproducibility among similar studies, which can be overcome through the standardization of the methods used for the decellularization process.

Considering the clinical trials status quo, although there are limited clinically approved tissue-engineered products, a rapid move toward more targeted therapies and customized treatments supported by 3D technologies has been noticed, particularly for cellular scaffold-based approaches.

Amongst the assortment of bioactive materials used for the production of 3D structures, composite materials appear to be the most promising ones. By combining polymer and ceramic biomaterials and simulating the natural tissues, better strength, adequate immune response, and biodegradability can be ensured. Although current research shows promising results, both from the mechanical and biological points of view, long-term studies are needed to ensure the implant-tissue interactions, resorption, and hierarchical structure, and finally to turn them into a clinically viable strategy. In this sense, some prospective improvements are under investigation to create enhanced TERM products, as demanded by regulatory approval bodies, before application in human patients. Improvement of cell-scaffold interaction with the use of cell-adhesive ligands, and changing cell morphology, alignment, and phenotype by functionalizing the surface of the scaffolds or even by mechanobiological stimulation of the cells open tremendous opportunities for regulation of TERM products.

## Figures and Tables

**Table 1:** The most recent studies of cellular and acellular 3D porous scaffold strategies for TE purposes.

Technology	Materials	Cells/Growth Factors	Outcomes	Application	Ref.
Freezing and lyophilization	Collagen (Col)/carbon nanotube (CNT)/chitosan (CS)/hydroxyapatite (HAp)	-	Increased hydrophilicity from 87.8° to 76.7° and improved mechanical properties of the composite scaffolds compared to Col (211 kPa), CS (284 kPa), Col/CNT (311 kPa), and Col/CNT/CS (524 kPa) scaffolds	Bone tissue engineering	[129]
	Na-alginate /hydroxyethylcellulose /HAp	-	After loading with Hap, the mechanical properties of the scaffolds increased deformation energy and rigidity gradient (19.44 ± 0.85 Pa), with bioactivity and biocompatibility in vitro and in vivo (implanted in femur of adult male Wistar rats for 6 weeks)		[80]
	Collagen from shark skin/ CaPs from shark teeth	Saos-2 cells seeding	Use of EDC/NHS crosslinking increased the attachment and proliferation of osteoblast-like cells		[82]
	Silk fibroin and β-tricalcium phosphate (TCP)	Human adipose stem cells (hASCs) seeded on the scaffolds	Highly interconnected macroporosity.; significant responses of hASCs proliferation and differentiation when varying the ionic dopants in the scaffolds		[64]
	Collagen and denatured collagen (DCol)	Rabbit chondrocytes seeding	Adhesion, proliferation, and re-differentiation of chondrocytes by Col scaffolds with triple helix and the regeneration of cartilage defects, compared with the DCol scaffolds	Cartilage tissue	[130]
	PLLA, PCL, and collagen type I	Adipose tissue-derived mesenchymal stem cells seeding	Mechanically stronger mesh support, provided by PCL-PLLA and cell adhesion, and tissue formation promoted by the collagen type I microsponges	Skin	[131]
	Silk fibroin	-	Elastic modules of the scaffolds between 100 and 900 kPa	n.d.	[132]
	Decellularized extracellular matrix (dECM)/gelatin/chitosan	rat BMSCs seeding	Enhanced elastic modulus, no cytotoxicity, and enhanced proliferation	Meniscus tissue	[17]
	bovine small intestinal submucosa (bSIS) layers/HAp microparticles/PCL	rat BMSCs seeding	Enhanced cell proliferation and osteoblastic differentiation within 21 days. Maximum strength similar in cell-laden scaffolds and cell-free scaffolds in wet conditions.	Bone	[19]
Robocasting	Biphasic CaP doped with Sr and Ag	MG-63 cells	Different pore sizes with compressive strengths comparable to cancellous bone. Sr and Ag improved the mechanical strength and cell proliferation and granted good antimicrobial activity against *Staphylococcus aureus* and *Escherichia coli*	Bone tissue engineering	[117]
	Biphasic CaP and chitosan	hDNFs (human dermal neonatal fibroblasts)	Produced levofloxacin loaded scaffolds without the sintering step. The antibiotic was not degraded during the fabrication process and its bactericidal efficacy was preserved		[119]
3D bioprinting	PCL and bioactive borate glass	hASCs-laden	Controlled release of bioactive glass; more than 60% viable hASCs on the scaffolds after 1 week of incubation.	Bone tissue engineering	[133]
	Polycaprolactone (PCL)	Saos-2 cells seeding	The non-orthogonal structures showed higher *E* moduli than the orthogonal one, with a positive influence on the biological performance of the cells; higher values for the mineralization, activity of osteogenic-related genes, and deposition of the mineralized matrix		[104]
	Alginate/alginate-sulfate	MC3T3-E1 cells/BMP-2	Alginate/alginate sulfate bioinks allowed good 3D cell printing. Improvement of the release of BMP-2 was achieved using alginate sulfate. Proliferation and differentiation of the printed osteoblasts were enhanced		[90]
	GelMA and methacrylated hyaluronic acid (HA) modified with HAp	hASCs	Positive effects on bone matrix production and remodelling		[134]
	Collagen/dECM/silk fibroin (SF)	MC3T3-E1 cells	High compressive modulus mainly due to the methanol-treated SF; high cellular activities in in vitro tests using MC3T3-E1 cells, induced by Collagen and dECM.		[135]
	α-TCP/collagen	MC3T3-E1 cells	The scaffold showed good mechanical properties and cellular activities		[128]
	collagen type I/agarose with sodium alginate	Primary chondrocytes	Addition of collagen or agarose had an impact on gelling behavior and improving mechanical strength. The collagen facilitated cell adhesion, accelerated cell proliferation, and enhanced the expression of cartilage-specific genes, (*Acan*, *Sox9*, and *Col2a1*)		[126]
	Fibrin and wollastonite	Loaded with rabbit BMSCs	Possible extensive regeneration of both cartilage and subchondral bone induced by in vivo transplantation of the scaffolds	Osteochondral tissue	[136]
	Collagen	MC3T3-E1	Cell-laden scaffold using tannic acid for crosslinking process. TA crosslinking increased mechanical properties and high cell viability	n.d.	[127]
	CS/PCL	dECM coating/WJMSCs seeding	Improved osteogenic differentiation in vitro and bone regenerative potential in vivo	Bone	[107]
	PCL/β-TCP	dECM coating/MC3T3-E1 seeding	Improved osteogenic differentiation in vitro and bone regenerative potential in vivo	Bone	[120]
Laser sintering technique	PCL and HAp	-	Subchondral bone regeneration and articular cartilage formation in a rabbit model	Osteochondral tissue	[137]
Sol-gel method combined with 3D plotting	HAp/chitosan/silica	Mouse BMSCs seeding	Compressive strength comparable to the human trabecular bone	Bone regeneration	[138]
BG obtained by sol-gel method	Zein/bioactive glass (BG)	MG-63 cells seeding	Ag-doped BG scaffolds showed antibacterial properties.		[139]
Electrospinning combined with electro-spraying	PCL/HAp	Murine embryonic cell seeding	High capacity to guide the migration of differentiated bone cells throughout the cavities and the ridge of the scaffolds		[140]
	PCL/gelatin and multi-walled carbon nanotubes (MWNTs)	Adult rabbit chondrocytes seeding	Increased hydrophilicity and tensile strength, and higher bioactivity and slower degradation rate due to presence of MWNTs;	Cartilage tissue	[141]
Electrospinning	Graphene-incorporated electrospun PCL/gelatin	PC12 cells	99% antibacterial properties against gram-positive and gram-negative bacteria. Good cell attachment and proliferation	Nerve tissue engineering	[142]
	PCL/collagen	Human endometrial stem cells seeding	Higher wettability, attachment, and proliferation rates of hEnSCs on the PCL/collagen scaffold	Skin	[143]
	Polyhydroxybutyrate-co-hydroxyvaletare (PHBV) containing bredigite	-	Bredigite nanoparticles increased the mechanical properties, biodegradability, and bioactivity of the scaffolds	Bone tissue	[144]
	PLLA/β-TCP	hMSCs seeding	Enhanced water uptake ability, in vitro bio-mineralization, and bioactivity promoted by the incorporation of β-TCP	Bone	[145]
	PCL/Silk fibroin (SF)	Human fibroblast seeding	Good tensile strength, elasticity, and increased degradation rate, as well enhanced cell proliferation, with the presence of SF	n.d.	[146]
Electrospinning combined with 3D bioprinting	PCL	Laden with L929 mouse fibroblasts	Multi-layered structures—3D scaffolds—with loosely packed nanofibers, with better surface wettability (when compared to the 2D scaffolds)	n.d.	[147]
Phase separation process	Cartilage ECM-derived/PLGA-β-TCP-collagen type I	BMSCs seeding	Enhanced OC regeneration. Chondro and osteogenic-induced BMSCs with independent environments	Osteochondral tissue	[148]

Note: n.d.: not defined; BMSCs: bone marrow stem cells; bSIS: bovine small intestinal submucosa; ECM: extracellular matrix; HA: hyaluronic acid; hMSCs: human mesenchymal stem cells; hASCs: human adipose stem cells; HAp: hydroxyapatite; PCL: polycaprolactone; PLLA: poly-L-lactic acid; SF: silk fibroin; TCP: tricalcium phosphate.

**Table 2:** Technologies and crosslinking methods for producing innovative hydrogel-based matrices for TE applications.

Technology	Materials	Crosslinking Method	Outcomes	Application	Ref.
Injectable hydrogels	Carboxymethyl chitosan and alginate integrated with HAp nanoparticles and calcium carbonate microspheres (CMs)	Chemical crosslinking between amino and aldehyde groups of carboxymethyl chitosan (CMCS) and oxidized alginate (OAlg)	Controlled gelation time, morphology, mechanical properties, swelling ratio, and in vitro degradation by varying HAp and CMs contents; sustained drug release and antibiotic activity against bacteria	Bone tissue engineering and drug delivery	[214]
	Poly(ethylene glycol)-N-hydroxysuccinimide (PEG-NHS)	Physical crosslinking using gelatin functionalized with norbornene groups (GelNB) and crosslinked with thiol-functionalized poly(ethylene glycol) (PEGdiSH) using a LAP initiator	Cell-laden ability inside the microgels formed as 3D constructs; human BMSC viability and function preservation within the structures; upregulation of chondrogenic activity and glycosaminoglycans (GAGs) formation encouraged by the assembled microgels	Articular cartilage regeneration	[215]
	Methacrylated decellularized cartilage hydrogel (MeSDCC) with HAp nanofibers (HAPnf), or bioglass (BG)	Photo-crosslinking	Increased mechanical stiffness and minimal bone regeneration in vivo	Bone regeneration	[178]
3D printing	Alginate (AL), Methylcellulose (MC), Halloysite Nanotube (HNT), and Polyvinylidene Fluoride (PVDF)	Chemical crosslinking using calcium chloride (CaCl_2_) after printing	High water content and good miscibility in the printed structures; chondrocyte viability after 4 days of culture increased by the presence of PVDF	Cartilage applications	[216]
	Sodium alginate (SA) and gelatin (Gel)	Chemical crosslinking induced after printing by soaking in CaCl_2_ and glutaraldehyde	The hydrogels showed high transparency and excellent fluidic properties; interconnected porous formation after 3D architecting according to pre-established operating parameters; 3D printed architectures allowed chondrocyte viability and proliferation with efficient distribution within the porous structures	Cartilage repair	[217]
	Ultrapure alginate and methylcellulose (Alg/MC)	Chemical crosslinking by Alg/MC blending	Successful encapsulation of pancreatic islets in hydrogels; good diffusion of glucose and insulin within the structures, with the embedded islets continuously producing insulin and glucagon, while still reacting to glucose stimulation	Pancreatic islets transplantation	[218]
	Polycaprolactone (PCL)/β-tricalcium phosphate (TCP)/bone decellularized ECM (dECM)	No crosslinking	The scaffolds compressive modulus ranged from 31.3 to 39.9 MPa, having excellent bone regeneration efficacy in vitro and in vivo	Bone tissue	[120]
Freeze-drying	Silk fibroin (SF) and sodium alginate (SA)	Ionic crosslinking utilizing Ca^2+^ from calcium silicate (CS) to simultaneously crosslink SF and SA	CS inside the porous SF/CS/SA hydrogel-based structures remarkably enhanced hydrophilicity, degradation, compression resistance, bioactivity and pH of structures; the presence of CS stimulated BMSCs proliferation and ALP activity at certain concentrations	Bone tissue engineering	[219]
	Gelatin (Gel) and polycaprolactone−polyethylene glycol (PCEC)	Chemical crosslinking of Gel solution with glutaraldehyde, incorporation PCEC nanoparticles added to the Gel solution	Gel porous hydrogels incorporating PCEC nanoparticles loaded with TGF-β presented a sustained release of the growth factor and positively affected structures porosity; nanocomposite hydrogels cultured with h-AD-MSCs expressed chondrogenic-related markers with potential for chondrocytes differentiation	Cartilage tissue engineering	[220]
Salt-leaching	Poly(ethylene glycol) (PEG) and sodium chloride (NaCl)	Physical crosslinking of functionalized PEG solution induced by a photoinitiator and irradiated with UV light; NaCl particles added to PEG precursor solution for porosity inducement	Microporous PEG-based hydrogels supported low blood glucose levels at earlier times and provided the restoration of normoglycemia	Pancreatic islets transplantation	[221]
	Poly(lactide-co-glycolide) (PLGA), magnesium hydroxide, and renal dECM	No crosslinking	Magnesium hydroxide and dECM alleviated the inflammatory response and activated cell morphogenetic behaviors, influencing cell attachment and differentiation; the scaffold promoted the reconstruction of glomerular structure in renal tissue, contributing to the full recovery of the nephrectomized kidney	Kidney tissue	[222]

Note: ALP: alkaline phosphatase; BMSCs: bone marrow stem cells; h-AD-MSCs: human adipose-derived mesenchymal stem cells; HAp: hydroxyapatite.

**Table 3:** Overview of the complete and ongoing clinical trials, in the last 5 years, using scaffolds and hydrogel strategies for tissue engineering and regeneration. Information obtained from https://clinicaltrials.gov/.

ClinicalTrials.Gov Identifier (NCT)	Date and Status	Study	Procedure	Patients Age	Follow-Up	Primary Outcomes
NCT01301664	2013–2016 Completed	Cartilage Tissue Engineering	Harvested human cartilage tissues from osteoarthritic patient during total knee arthroplasty surgery	30–70 years	n.d.	n.d.
NCT01791062	2013–2016 Completed	Safety and Efficacy Study of HYTOP® in the Treatment of Focal Chondral Defects	Focal chondral defect in femoro-tibial compartment of the knee joint	18–50 years	12 weeks	Adverse events with causal relationship to the investigational medical device evaluated with respect to type, incidence, and intensity up to study termination of each subject
NCT01879046	2013–2017 Completed	Regenerative Medicine of Articular Cartilage: Characterization and Comparison of Chondrogenic Potential and Immunomodulatory Adult Mesenchymal Stem Cells	Total Knee arthroplasty	≥18 years	3 years	Increased expression of chondrogenic markers
NCT01813188	2013–2017 Completed (Phase 2)	Use of bone marrow mononuclear cells seeded onto a porous matrix of tricalcium phosphate ceramic and demineralized bone matrix, for the consolidation of tibial bone defects (pseudoarthrosis)	Autologous bone graft	18–75 years	6 months	Time needed to repair the focus of necrosis measured by pain radiography
NCT02033226	2014–2015 Completed (Phase 3)	Evaluation of Clinical, Anti-Inflammatory, and Anti-Infective Properties of Amniotic Membranes Used for Guided Tissue Regeneration in Contained Defects	Chronic Periodontitis	30–55 years	6 months	The mean difference in levels of hBD-2/IL-1β
NCT01362413	2014–2015 Completed	Validation of Laboratory Test for Predicting Bone Tissue Regeneration (Rebone-test)	Nonunion of Fracture (Pseudarthrosis)	≥ 18 years	12 months	Correlation between laboratory results at the surgery and clinical and radiographic results at 12 months, when patients will be considered as healed or not healed
NCT02409628	2015–2017 Completed	EktoTherix™ Regenerative Tissue Scaffold for Repair of Surgical Excision Wounds	Application of the EktoTherix scaffold to a fresh wound created by the surgical removal of non-melanoma skin cancers	≥18 years	3 months	Incidence of device related adverse events
NCT02513368	2015 Completed (Phase 2)	Peri Implant Soft Tissue Healing in Single Implant Restoration Using Two Different Techniques	Augmentation procedure with Bio-Oss® and Bio-Gide®	18–75 years	1 year and 1 month	Change from baseline in the clinical characteristics of the peri implant mucosa
NCT00900718	2016 Completed	Comparison of Straumann Bone Ceramic and Bio-Oss With Guided Tissue Regeneration for Alveolar Ridge Preservation	Bone augmentation, after tooth extraction	18–75 years	32 weeks	The changes of bone level between baseline and 32-weeks post-extraction
NCT02859025	2016 Completed (Phase 1)	Concomitant Use of Buccal Fat Pad Derived Cells and Autogenous Bone in Alveolar Cleft Osteoplasty	Cleft of Alveolar Ridge	Child, adult and older adult	6 months	Change in bone volume
NCT03113747	2017 Completed (Phase 1–2)	Allogeneic Adipose derived stem cells (ADSCs) and Platelet-Poor Plasma Fibrin Hydrogel to Treat Patients with Burn Wounds	Application over perforated autologous skin graft following the covering with hypoadhesive bandage	18–65 years	1 month	The degree of healing of skin flap after autologous skin grafting
NCT03076138	2017-2019 Completed	Gene-activated Bone Substitute for Maxillofacial Bone Regeneration	Bone grafting with gene-activated matrix maxillofacial regeneration	18–60 years	6 months	Bone tissue formation in the field of gene-activated bone substitute implantation
NCT01605201	2018 Completed (Phase 1)	Tissue Engineered Nasal Cartilage for Regeneration of Articular Cartilage (Nose2Knee)	Implantation of a graft in a degenerative lesion of articular cartilage of knee	18–55 years	24 months	Safety for the patient and stability of the graft
NCT02673905	2018 Recruiting	Clinical Trial for the Regeneration of Cartilage Lesions in the Knee (NosetoKnee2)	Articular cartilage lesions in the knee	18–65 years	24 months	Comparison of the efficacy of the two investigational medicinal products (IMPs)
NCT02145130	2018 Recruiting (Phase 1)	Phase I Study for Autologous Dermal Substitutes and Dermo-epidermal Skin Substitutes for Treatment of Skin Defects	Transplantation of an autologous tissue-engineered dermal substitute	1–70 years	21 days	Assessment and reporting of local infection rate and graft take
NCT03613090	2019 Not yet recruiting (Phase 2)	Novel Collagen Scaffold versus Conventional Scaffold in Regeneration of Human Dental Pulp Tissue	FDA-approved collagen-hydroxyapatite material called Syn-Oss for regeneration of pulp tissue	≥12 years	15 months	Observation of: Radiodensity at apex at 1mm from root vertex; increase in dentin wall thickness; increase in root length, in mm, and Periradicular status
NCT02090140	2015–2020 Ongoing	Microfracture Versus ADSCs for the Treatment of Articular Cartilage Defects	Microfracture followed by the application of ADSCs to the defect site	18–50 years	6, 12, 24 months	Health Scores on the Knee injury and Osteoarthritis Outcome Score (KOOS) Questionnaire
NCT01765244	2013–2020 Ongoing (Phase I–II)	Allogeneic Tissue Engineering (Nanostructured Artificial Human Cornea) in Patients with Corneal Trophic Ulcers in Advanced Stages, Refractory to Conventional (Ophthalmic) Treatment	Implantation of an anterior lamellar nanostructured artificial human cornea with allogeneic cells from dead donors embedded in a fibrin-agarose scaffold	≥18 years	24 months	Adverse events (and serious adverse events) causally related to experimental treatment; implant status (integrity, detachment, and reabsorption); local, regional, or systemic infections related with the implant; induced corneal neovascularization
NCT03698721	2018–2026 Ongoing	Urothelium Tissue Engineering Using Biopsies from Transurethral Resection of Prostate	Transurethral Resection of Prostate	≥18 years	6, 12, 36 months	Histological analysis of biopsy
NCT03103295	2018 Ongoing (Phase 1–2)	3D Tissue Engineered Bone Equivalent for Treatment of Traumatic Bone Defects	Tissue-engineered bone-like construct transplantation	18–60 years	12, 36 months	Radiographic and MRI assessment in progression

Note: n.d.: not defined; ADSCs: adipose-derived stem cells.
